# From the Editor’s Desk

**Published:** 2023

**Authors:** Professor PJ Commerford

**Figure:**
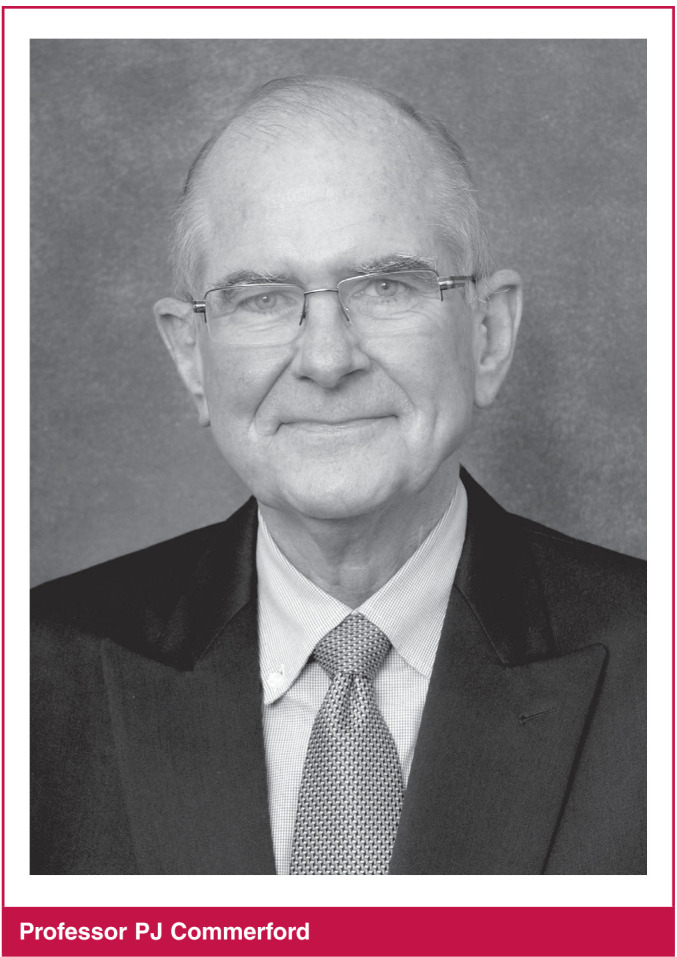
Professor CJ Commerford

Clinicians often face the tough task of deciding, and conveying those decisions to patients and their families, about whether or not to introduce anticoagulants when a diagnosis of atrial fibrillation has been made. The established benefits of wellcontrolled warfarin therapy and novel oral anticoagulants (NOACS) are clear. Despite clear guidelines, there remains a reluctance on the part of some clinicians and patients to embrace these guidelines and the risk of bleeding remains a concern. The published guidelines define the bleeding risks associated with anticoagulant use.

This issue of CVJA has an intriguing article by Wu and colleagues (page 231). They provide a retrospective report on the application of computed tomographic angiography and echocardiographic imaging in predicting left atrial appendage (LAA) thrombosis in patients with non-valvular atrial fibrillation. They demonstrate that both imaging modalities had high predictive values for the presence LAA thrombosis in the patients studied. It is unlikely that such imaging will become routine in clinical practice but it may have a role in patients at high risk of bleeding, in selecting candidates for implantation of a LAA occluder device.

In another retrospective study, Duzyol and Şaşkin (page 198) explore the association between pre-operative carotid intima– media thickness (CIMT) and early postoperative acute kidney injury (AKI) following isolated coronary artery bypass grafting (CABG). Increased CIMT has been proposed as a quantitative index of subclinical atherosclerotic disease progression and a surrogate measure for cardiovascular disease. Pre-operative CIMT was found to be an independent predictor of AKI in the early postoperative period after CABG. In this instance, again, it is unlikely that carotid imaging will become routine in this population but it may be helpful in selecting patients for CABG, particularly in resource-constrained settings where access to dialysis may not be easily available.

In a case report, Lin and co-authors (page 256) draw our attention to Kounis syndrome. It was thought to be a rare condition but is now being more commonly identified as the cause of acute coronary events in patients without a previous history of coronary artery disease. Kounis syndrome is the occurrence of acute coronary syndrome after an allergic insult, which can be hypersensitivity, or anaphylactic or anaphylactoid conditions. The underlying mechanism has been attributed to mast cell and platelet activation and interaction with other inflammatory cells such as macrophages and T lymphocytes, with involvement of a range of cytokine cascades. The end effect is coronary artery spasm and/or atheromatous plaque erosion or rupture. To date, three variants have been proposed, coronary spasm, acute myocardial infarction and stent thrombosis.

